# Case report: Unusual breast cancer metastasis manifesting as a scalp lesion in a patient with invasive lobular carcinoma

**DOI:** 10.3389/fonc.2024.1361333

**Published:** 2024-04-05

**Authors:** Nam Hee Koh, Ha Yeun Oh

**Affiliations:** Department of Radiology, Kangwon National University Hospital, Chuncheon, Republic of Korea

**Keywords:** distant metastasis, cutaneous metastasis, scalp metastasis, invasive lobular carcinoma of the breast, metastatic breast cancer, scalp nodule

## Abstract

Breast cancer is the most prevalent cancer in women globally, often leading to distant metastasis in the lung, liver, or bones. Cutaneous metastasis represents an uncommon pattern in breast cancer, but when observed, it tends to manifest in the thorax and upper abdomen, primarily due to lymph node involvement. Therefore, occurrences of cutaneous metastasis on the scalp and extremities are infrequent. Moreover, invasive lobular carcinoma metastasizing to remote skin is rare among the breast cancer. This report presents a case of cutaneous metastasis of invasive lobular carcinoma to the scalp in a patient treated for breast cancer six years ago, with no signs of local recurrence or metastasis to other organs.

## Introduction

Breast cancer is globally recognized as the most common cancer in women, with recent trends indicating an increase in both incidence and mortality rates worldwide (1). With advancements in diagnostic technologies, developed countries have witnessed a decline in breast cancer mortality due to early detection and treatment. Paradoxically, however, statistical evidence suggests a rising incidence of metastatic breast cancer (2).

Metastasis in breast cancer predominantly occurs in specific organs, with rare instances of cutaneous metastasis. Although cutaneous metastasis from breast cancer is uncommon, accounting for approximately 2.5%, it stands out as a relatively proficient entity compared to the less than 1% occurrence in general solid tumors. As a result, breast cancer demonstrates a heightened propensity for cutaneous metastasis, accounting for approximately 33% of all cases among metastatic cancers affecting the skin (3, 4).

Cutaneous metastasis primarily occurs in proximity to the primary tumor site, occasionally leading to misinterpretation as a secondary malignancy related to the recurrence or treatment of the primary lesion. Moreover, when tumors appear on the scalp or extremities far from the primary lesion, linking them to breast cancer becomes more challenging.

This case report discusses a rare scenario where a scalp nodule, incidentally discovered during the surveillance period after breast cancer treatment, was confirmed and treated as a distant metastatic lesion of breast origin through nuclear imaging and histopathological examination. The findings highlight the importance of vigilance in identifying remote metastases manifesting as a skin lesion in breast cancer survivors and underscore the need for comprehensive monitoring strategies.

## Case presentation

**Figure 1:**
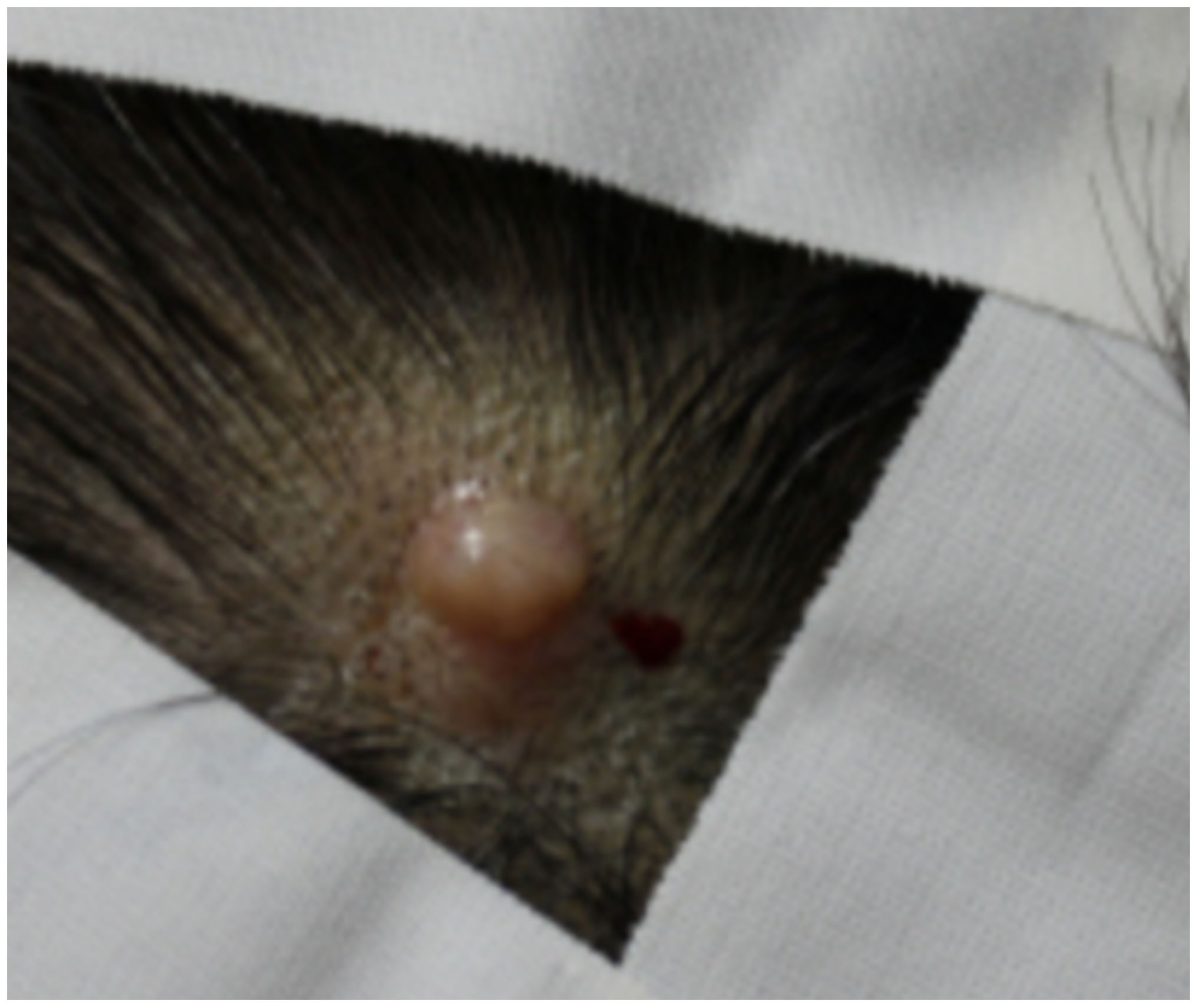
Clinical image of the scalp lesion. A pinkish-hued, 2 cm-sized nodular lesion is observed, accompanied by localized alopecia at the left parietal area of the scalp.

A 54-year-old female patient presented to the dermatology clinic due to the enlargement of a scalp nodule in the left parietal area. The lesion, measuring approximately 2 cm and displaying a pinkish hue, appeared as a nodular lesion accompanied by localized alopecia (**Figure 1**). According to the patient’s account, she first noticed the lesion five years ago during chemotherapy for breast cancer, experiencing overall hair loss due to the side effect of the treatment. However, since the lesion was small and did not grow, she did not seek further medical attention at that time.

The patient was diagnosed with invasive lobular carcinoma six years ago, located in the upper outer quadrant of the left breast. At the time of diagnosis, Positron emission tomography with 2-deoxy-2-[fluorine-18]fluoro-D-glucose integrated with computed tomography (^18^F-FDG PET/CT) showed no evidence of distant metastasis. Following left breast-conserving surgery, the postoperative pathological assessment revealed a pT3N1c classification, indicating the presence of cancer cells spreading to the left axillary lymph nodes. Additionally, the tumor tested positive for estrogen and progesterone receptors, and lymphovascular invasion was observed. Subsequently, she underwent adjuvant chemotherapy with the doxorubicin, cyclophosphamide, and docetaxel regimen for six cycles, followed by oral tamoxifen and radiation therapy targeting the left breast and axilla.

Over the course of six years of regular follow-up examinations, there was no evidence of recurrence or distant metastasis. The patient continued oral tamoxifen therapy. The most recent follow-up examination, four months before the dermatology visit for the scalp nodule, also showed no signs of recurrence or distant metastasis.

**Figure 2:**
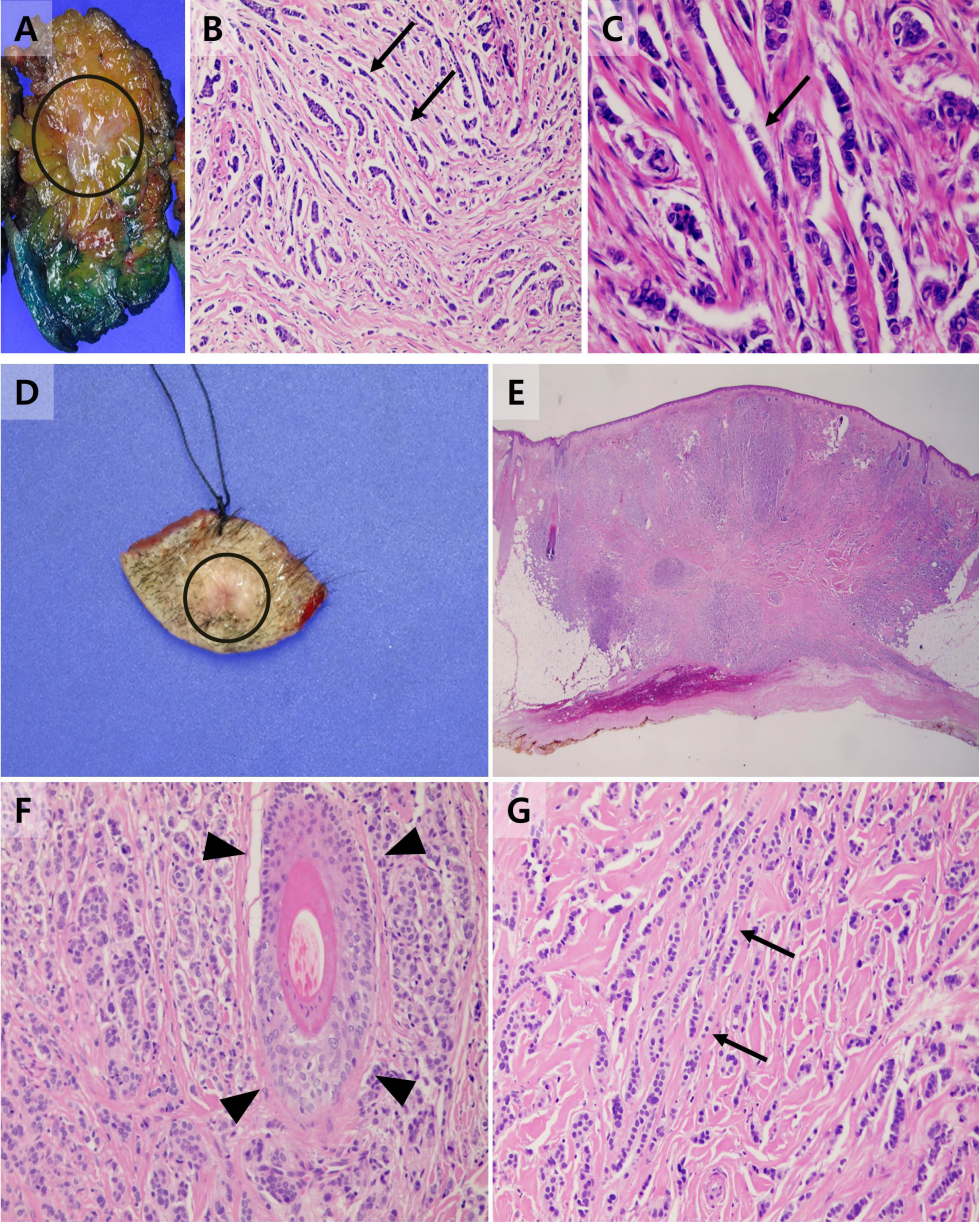
Gross specimen and histopathological findings of the primary breast cancer and scalp lesion. **(A)** A gross specimen of the primary breast cancer diagnosed invasive lobular carcinoma with the area involved by the primary tumor annotated as a circle. **(B)** Tumor cells of the primary invasive lobular carcinoma arranged in slender linear strands one to two cells across, exhibiting the so-called Indian file appearance (arrow) (HE x 200). **(C)** On high-power photomicrograph, tumor cells are also arranged in single files, cords, and single cells (arrow) (HE x 400). **(D)** A gross specimen of the scalp lesion with the area involved by the metastasis annotated as a circle. **(E)** On low-power photomicrograph, tumor cells infiltrated the subcutaneous layer of the scalp without evidence of destruction to adjacent structures or skin appendages. (HE x 15) **(F)** Intact skin appendages were observed in high-power photomicrographs (arrowhead) (HE x 200) **(G)** Tumor cells of the scalp tumor showed a similar structural pattern to the primary breast cancer, exhibiting an Indian file appearance (arrow) (HE x 200).

**Figure 3:**
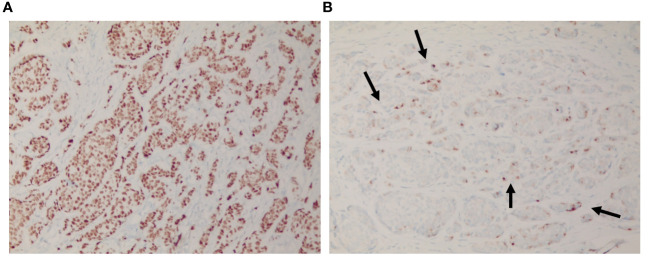
Immunohistochemical findings of the scalp lesion. **(A)** The immunohistochemical analysis revealed a positive staining for estrogen receptor, and **(B)** scattered partial positive staining for GCDFP-15.

Considering the patient’s medical history, a punch biopsy was performed to rule out the possibility of cutaneous metastasis. The tissue sample obtained from the punch biopsy exhibited structural features similar to those of previously diagnosed breast cancer tissues. Immunohistochemical staining showed positivity for cytokeratin 7 (CK7), estrogen receptor and partial positivity for gross cystic disease fluid protein 15 (GCDFP-15), a breast cancer-specific marker (**Figures 2**, **3**). In light of these cumulative observations, there arose a suspicion that the scalp lesion might be indicative of metastasis originating from breast cancer.

**Figure 4:**
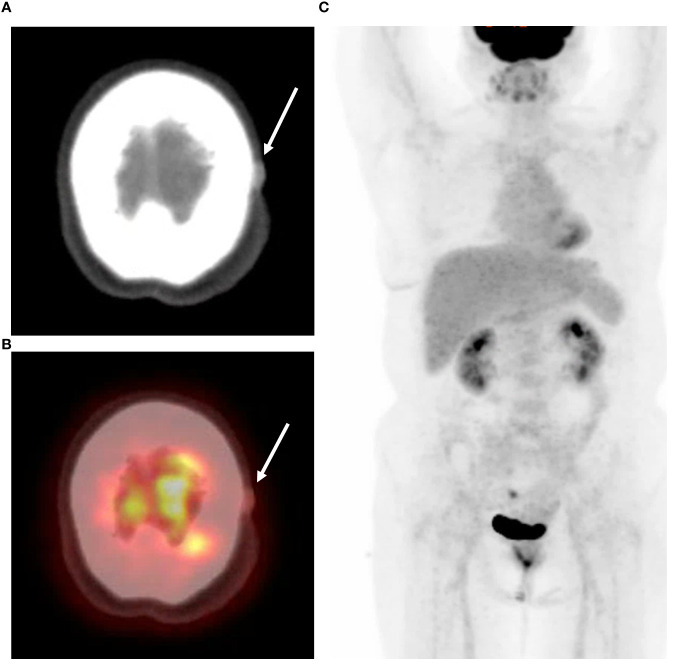
PET-CT findings of the patient. **(A)** A 1.5cm sized bulging lesion with soft tissue attenuation, involving subcutaneous layer of scalp along the left parietal area. **(B)** This lesion had a measured SUVmax value of 2, indicating a slight degree of hypermetabolism. **(C)** Maximum intensity projection images of PET-CT showed no evidence of other distant metastasis or recurrence.

Subsequent ^18^F-FDG PET/CT imaging revealed slightly increased FDG uptake in the lesion. No evidence of metastasis to other organs, excluding the scalp, or recurrence at the previously treated primary site was observed (**Figure 4**).

The patient underwent excision of the scalp lesion, and the final pathology confirmed it as a metastatic lesion. Although adjuvant radiation therapy for scalp lesion and gonadotropin-releasing hormone agonist therapy were recommended, the patient declined, opting only to continue oral anastrozole therapy. Subsequent follow-up examinations did not reveal any signs of recurrence or distant metastasis, including in the breast and scalp.

## Discussion

The metastasis of breast cancer is not a random occurrence; it can be explained by the concept of organotropic metastasis, where certain organs are more susceptible to metastasis (5). Commonly known sites for metastasis include bones, liver, brain, lungs, and distant lymph nodes (6). This multifaceted phenomenon results from a combination of factors such as the subtype of the primary tumor, the molecular and genetic characteristics of breast cancer, and the interactions between metastatic organs and cancer cells (7). In this context, it is not surprising that the metastatic patterns differ between invasive ductal carcinoma (IDC) and invasive lobular carcinoma (ILC). As mentioned above, since IDC constitutes the majority of breast cancers, metastases of breast cancer often appear common in the lungs, liver, and bones. However, in the case of ILC, it is known to have a higher prevalence of metastasis to the gastrointestinal tract, gynecologic organs, peritoneal surface, or retroperitoneum due to its distinct pattern of E-cadherin protein expression (8, 9).

Nevertheless, cutaneous metastasis in breast cancer is considered rare in case of both IDC and ILC. According to a study analyzing the metastatic patterns of breast cancer, skin metastasis accounted for 7.1% in IDC and 6.5% in ILC among all metastatic sites, respectively (10). However, it comprises approximately 33% of cutaneous metastatic lesions, underscoring breast cancer’s propensity for cutaneous metastasis compared to other malignancies (3, 4).

The majority of breast cancer cutaneous metastases are found in close proximity to the primary tumor, suggesting that metastasis through local lymph node drainage plays a significant role (6). Consequently, cutaneous metastasis in breast cancer predominantly manifests in the chest wall and upper abdomen (11). Meanwhile, the findings of distant skin metastasis far from the primary lesion (i.e. upper/lower extremities or scalp) still remain questionable. There was no specific paper that precisely explained the mechanism of distant skin metastasis from primary lesions yet. However, by examining the molecular pathology of ILC and the metastatic patterns of both ILC and IDC, a novel insights into the occurrence of cutaneous metastasis distant from the primary lesion could potentially be gained. Some studies reveals that the incidence of cutaneous metastasis is more frequent in ILC than IDC due to the characteristic of loss of E-cadherin (12–14). Among the ILC, certain types exhibit a heightened tendency for metastasis to other organs, believed to be linked to changes in intracellular protein or hormonal receptor expression, as well as alterations in signaling pathways due to molecular alterations (12).

The patterns of cutaneous metastasis in breast cancer are highly diverse, with normochromic papules or nodules being the predominant forms (15). Particularly when occurring on the scalp, these lesions often present as tumor-associated hair loss, where nodules or hardened plaques lead to irreversible alopecia due to destruction of hair follicles by the primary tumor (16). Lesions of metastatic breast cancer that arise on the scalp occasionally require discrimination from appendage tumors such as eccrine spiradenoma, which are cutaneous appendage-related (17). In such cases, the utility of estrogen receptors is limited, as sweat glands, akin to breast tissue, can express estrogen receptors (18). However, the majority of breast cancers typically show positivity for CK7 and negativity for CK20, making immunostaining with these markers helpful for differentiation (19). Furthermore, GCDFP-15 is known as an immunohistochemical marker frequently used when there is a suspicion that a tumor of unknown primary origin may be of mammary origin, given its specificity for mammary differentiation (20). Of greater significance, the pathological specimen of the scalp lesion exhibited structural features akin to the previously diagnosed breast cancer, with malignant lesions infiltrating surrounding tissues without evident destruction of cutaneous appendages. These findings suggest not so much a primary malignancy originating from cutaneous appendages but rather indicate a metastatic lesion from a past history of breast cancer.

In many cancer types, cutaneous metastasis implies widespread dissemination, making complete resection unfeasible and necessitating palliative and supportive care (21). However, in breast cancer, skin lesions tend to appear regardless of the cancer’s stage, occasionally leading to the diagnosis of primary breast cancer through cutaneous metastatic lesions (22–24). Therefore, when diagnosing skin lesions in patients with a history of breast cancer, considering the breast cancer history is imperative. Presently, various types of breast cancers including ILC can be classified beyond the microscopic level to molecular subtypes (12). It is imperative to consider that specific subtypes with a heightened tendency for distant metastasis due to molecular-level mutations may exhibit divergent responses to commonly employed therapeutic agents when formulating a treatment plan. Additionally, recognizing that cutaneous metastasis in breast cancer does not always signify extensive dissemination emphasizes the need for personalized and tailored treatments based on the individual patient’s condition.

## Data availability statement

The original contributions presented in the study are included in the article/supplementary material. Further inquiries can be directed to the corresponding author.

## Ethics statement

The studies involving humans were approved by Institutional Review Board of Kangwon National University Hospital. The studies were conducted in accordance with the local legislation and institutional requirements. Written informed consent for participation was not required from the participants or the participants’ legal guardians/next of kin in accordance with the national legislation and institutional requirements. The written informed consent for publication was not obtained due to the retrospective and anonymized nature of the case report. All identifying patient information has been sufficiently anonymized to protect confidentiality. The study adheres to ethical standards, and the retrospective design precluded obtaining individual consents. Additionally, the authors have submitted an exemption request to the institutional review board of their affiliated institution, and the content has been approved. The manuscript strictly follows ethical guidelines and respects patient privacy, ensuring that no identifiable information is disclosed. Written informed consent was obtained from the participant/patient(s) for the publication of this case report.

## Author contributions

NK: Writing – original draft. HO: Writing – review & editing.
